# Impact of Iron Speciation on the Cytotoxicity and Gene Expression Profile of Biofortified *Hericium erinaceus* in Human Colorectal Adenocarcinoma

**DOI:** 10.3390/molecules31132295

**Published:** 2026-07-01

**Authors:** Klaudia Słyszyk, Kamila Rachwał, Ewa Baranowska-Wójcik, Dominik Szwajgier, Jan Sadurski, Adam Waśko

**Affiliations:** Department of Biotechnology, Microbiology and Human Nutrition, University of Life Sciences in Lublin, Skromna 8, 20-704 Lublin, Poland; klaudia.kowalik@up.edu.pl (K.S.); kamila.rachwal@up.edu.pl (K.R.); ewa.baranowska@up.edu.pl (E.B.-W.); dominik.szwajgier@up.edu.pl (D.S.); jan.sadurski@up.edu.pl (J.S.)

**Keywords:** *Hericium erinaceus*, iron biofortification, colorectal cancer, HT-29 cells, iron speciation, FeHBED, gene expression, molecular mechanisms, functional food

## Abstract

Colorectal cancer (CRC) is a major global health threat, necessitating the development of functional foods with chemopreventive potential. This study aimed to evaluate the modulation of the anticancer activity of *Hericium erinaceus* with different iron forms (FeCl_3_, FeSO_4_, and FeHBED) as it modulates its anticancer activity against HT-29 cells. The extracts were subjected to simulated in vitro digestion and analyzed for cytotoxicity (MTT), antioxidant capacity (DPPH), and morphological changes, alongside high-throughput RT-qPCR profiling of 92 cancer-related genes. The results demonstrated that iron speciation is critical, with FeHBED-biofortified extracts exhibiting the most potent concentration-dependent cytotoxic and antiproliferative effects. Treated cells displayed apoptotic morphology, including chromatin condensation and cell shrinkage. Molecular analysis revealed significant downregulation of key oncogenes (HRAS, MYC), cell cycle regulators (CDK4), and migration markers (RHOA, ITGB1), whereas VEGFA was upregulated as a stress-induced response. In conclusion, biofortification with FeHBED significantly enhanced the anticancer potential of *H. erinaceus* by targeting specific proliferative and survival pathways. These findings highlight the potential of iron-biofortified mushrooms as a functional dietary component for colorectal cancer prevention.

## 1. Introduction

According to the latest data from the World Health Organization (WHO), there has been a significant increase in the number of malignant tumors. They are currently the second leading cause of death worldwide and represent one of the greatest health challenges of the 21st century. Colorectal cancer is one of the most commonly diagnosed malignant tumors and poses a serious threat to public health on a global scale. In 2020, nearly 1.9 million new cases were reported and almost 935,000 deaths were attributed to this disease [1]. Despite significant advances in the treatment of colorectal cancer in recent years, patient survival rates remain insufficient. This is associated with the presence of unfavorable prognostic factors, such as vascular invasion, a low lymphocyte-to-monocyte ratio, and late detection of the disease. In response to these limitations, personalized medicine, which takes into account the individual characteristics of the patient, genomic profile, and biomarkers, is becoming increasingly important, allowing for more accurate selection of therapy and earlier intervention in people at risk [2].

For this reason, preventive healthcare and a diet based on a variety of products with proven health benefits play an important role. Particular attention should be paid *Hericium erinaceus* (Bull.) Persian, which shows promising biological potential. *H. erinaceus*, commonly known as Lion’s Mane, is valued for its medicinal properties, which result from the presence of numerous bioactive compounds. These substances are being intensively studied for their potential health benefits, in particular their neuroprotective, antioxidant, and immunomodulatory effects. The most important compounds responsible for these effects include erinacines, hericenones, polysaccharides, and other secondary metabolites [3]. Erinacins and hericenones affect neurotrophic pathways by stimulating the expression of nerve growth factor (NGF) while also exhibiting strong anti-inflammatory and antioxidant effects. As a result, they contribute to increased protection of nerve cells against oxidative stress and damage [4]. Studies indicate that the neuroprotective effect of erinacines is associated with the activation of the Nrf2 pathway and the regulation of genes responsible for the protection and regeneration of neurons, which has been confirmed in vitro and in vivo [5]. These effects may be further supported by the modulation of the gut–brain axis, as *H. erinaceus*. s Supplementation has been shown to increase the diversity of the gut microbiota and the abundance of bacteria that produce short-chain fatty acids, which promotes gut protection and reduces inflammation [6]. Research indicates that bioactive compounds in *H. erinaceus* may influence key mechanisms regulating the growth and survival of cancer cells, showing potential in the context of colorectal cancer. This effect is associated, among other things, with the inhibition of the PI3K/AKT/mTOR pathway and the reduction in epithelial–mesenchymal transition (EMT) [7]. Polysaccharides, including β-glucans present in Lion’s Mane, exhibit antioxidant, immunomodulatory, and anticancer properties [8]. The strong antioxidant and anti-inflammatory properties of *H. erinaceus* result from the presence of numerous phenolic and flavonoid compounds, and their activity depends on the growth stage of the mushroom. These compounds contribute to the reduction in oxidative stress and inflammatory processes, which play an important role in the development of chronic diseases [9].

In light of these beneficial properties, there is growing interest in strategies aimed at enhancing the content and bioavailability of health-promoting compounds, particularly through the development of functional foods, which can be further improved using approaches such as biofortification. Biofortification involves deliberately increasing the nutrient content of food and is closely related to their bioavailability. Unlike conventional fortification, it can lead to better absorption of minerals by increasing their concentration, modifying their chemical forms to make them more absorbable, and adding compounds that promote absorption, such as polyphenols. The promotion of biofortification is an important strategy for combating hidden hunger and improving nutrition, especially in regions with limited access to a varied diet. In this context, the biofortification of mushrooms is a particularly promising and innovative direction due to their ability to accumulate macro- and micronutrients [10]. For example, selenium plays an important role in protecting against disease owing to its antioxidant properties, while zinc is essential for the proper functioning of the immune and nervous systems. In turn, iron-biofortified mushrooms can be an effective aid in preventing iron deficiency, which is one of the most common nutritional problems worldwide [11].

Among edible mushrooms, Lion’s Mane has been identified as a promising candidate for biofortification due to its capacity to accumulate selected elements such as selenium, lithium, and iron. Studies have shown that *H. erinaceus* effectively accumulates selenium from the substrate and converts it into bioavailable organic forms. Among the selenium compounds studied, selenomethionine showed the strongest stimulating effect on fungal growth [12]. The results of this study indicate that biofortification of mushrooms with lithium, especially in the form of chloride, enables effective accumulation of this element without negatively affecting the growth or morphological characteristics of the fruiting bodies. Among the analyzed species, *H. erinaceus* showed the greatest bioaccumulation potential, and the lithium concentrations obtained suggest the possibility of covering a significant part of the recommended daily intake. These results justify further research on the bioavailability, safety, and potential use of biofortified Lion’s Mane as a functional source of lithium in the diet [13]. Studies have shown that the ability of *H. erinaceus* to accumulate selenium is strongly dependent on the strain, type of substrate, and cultivation conditions. Moderate levels of Se supplementation promoted both effective bioaccumulation and improved yields, with selenomethionine being the dominant form of selenium in most strains. The results confirm the possibility of using biofortified lion’s mane mushroom as a functional source of selenium and emphasize the importance of substrate selection and cultivation parameters in optimizing the content of this element [14]. Biofortification with various forms of iron increases its accumulation and affects the metabolism of the mushroom, with different sources of Fe producing different biological effects. These results confirm the possibility of enriching *H. erinaceus* with iron while maintaining its health-promoting properties [15].

Iron is a key element in the human body, playing an important role in various biological processes. Iron plays a fundamental role in the body, participating in the transport and storage of oxygen and in the energy processes occurring in the mitochondria. It is also essential for the proper functioning of enzymes involved in cellular metabolism, DNA synthesis, and the maintenance of genetic stability [16]. In addition, this element significantly influences the immune response by modulating the activity of cells in the innate and acquired immune systems [17].

Alterations in iron homeostasis may result in ferroptosis, a regulated form of cell death dependent on iron and lipid peroxidation, which plays an important role in the elimination of cancer cells. This process results from iron homeostasis disorders, increased oxidative stress, and antioxidant mechanism failure, and is a promising target in modern anticancer strategies. It manifests itself in the form of redox imbalance, increased concentrations of reactive oxygen species, and characteristic mitochondrial changes, such as damage to the outer membrane, matrix thickening, and reduction or loss of mitochondrial cristae [18]. Polyunsaturated fatty acids (PUFAs) in cell membranes are particularly susceptible to peroxidation, which is facilitated by enzymes such as long-chain acyl-CoA synthetase family 4 (ACSL4) and arachidonic acid lipoxygenases [19]. Glutathione peroxidase 4 (GPX4) plays a key role in protecting cells from ferroptosis by neutralizing lipid peroxides, while its inhibition results in their accumulation and induction of ferroptosis [20]. Non-coding RNAs (ncRNAs) regulate ferroptosis by controlling the expression of genes related to iron metabolism and antioxidant mechanisms, thereby influencing the susceptibility of cancer cells to this type of cell death [21]. Despite the promising therapeutic potential of ferroptosis, this process plays a dual role in cancer. On the one hand, it can limit tumor growth, while on the other hand, it can promote progression and weaken the immune response. For this reason, a full understanding of the mechanisms of ferroptosis and its links to other cell death pathways is essential for the development of effective and safe anticancer strategies, which requires further research before clinical application [22].

The aim of this study was to investigate the effect of Lion’s Mane extracts biofortified with iron in three different forms on the viability and morphology of colorectal cancer cells and on selected molecular mechanisms of cancer. This study is a continuation of the authors’ previous work on the biofortification of *H. erinaceus*.

The innovative nature of this research deserves special attention, as no studies analyzing the biological properties of iron-biofortified Lion’s Mane or the effect of different forms of this element on anticancer activity have been published to date.

The extracts were subjected to in vitro digestion, which allowed for the assessment of their potential bioavailability and biological activity after digestive processes. It was assumed that biofortification with iron in various forms could modulate the biological properties of *H. erinaceus*, leading to a reduction in cancer cell viability, morphological changes, and the regulation of molecular pathways, oxidative stress, and cell death.

The results obtained provide new data on the effect of biofortified natural products on colon cancer cells and indicate the potential preventive use of *H. erinaceus* while emphasizing the need for further in vivo studies, including clinical trials in humans.

## 2. Results

### 2.1. Cytotoxicity

**Figure 1:**
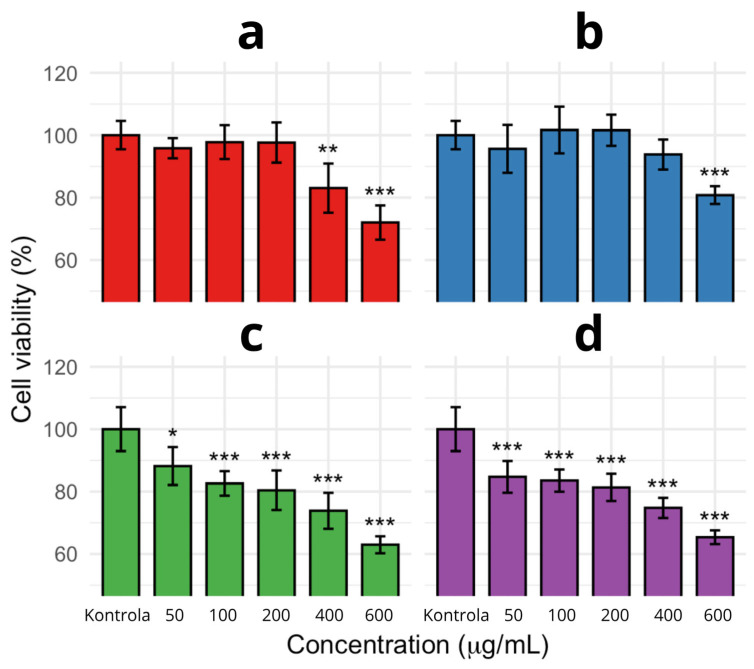
Effect of *Hericium erinaceus* extracts on the viability of HT-29 cells after 24 h, assessed by the MTT assay. The extract obtained from fruiting bodies biofortified with iron in the three forms of: FeCl_3_·6H_2_O (**b**); FeSO_4_·7H_2_O (**c**) and FeHBED (**d**) was compared with the extract from non-biofortified (untreated with any form of iron) fruiting bodies (**a**). Results are expressed as percentage of control (100% viability). Statistical significance: * *p* < 0.01, ** *p* < 0.005, *** *p* < 0.001; one-way ANOVA followed by Tukey’s post hoc test (*n* = 3 independent experiments).

In the conducted experiment, the in vitro digested extract was evaluated on HT-29 cells at concentrations of 50, 100, 200, 400, and 600 µg/mL. The cytotoxicity analysis performed using the MTT assay demonstrated that all tested extracts affected cell viability in a concentration-dependent manner; however, both the nature and magnitude of the effect varied depending on the applied biofortification method. The extract of *H. erinaceus* without biofortification exhibited moderate cytotoxicity, with a pronounced decrease in cell viability observed mainly at higher concentrations (400–600 µg/mL). In contrast, the extract biofortified with FeCl_3_·6H_2_O showed comparable or even higher cell viability than the non-biofortified sample, indicating no cytotoxic effect on the tested cells. Biofortification with FeSO_4_·7H_2_O resulted in a more pronounced cytotoxic effect, characterized by a gradual, dose-dependent decrease in cell viability, which was more evident than in the case of FeCl_3_·6H_2_O and the non-biofortified extract. The most significant changes were observed for the extract biofortified with FeHBED, which exhibited the strongest cytotoxic activity among all tested variants. A substantial reduction in cell viability was already evident at the lowest tested concentration, and this effect intensified with increasing concentration, reaching the lowest values at the highest doses. Figure 1 shows the results of the experiment.

These findings indicate that FeHBED significantly enhances the biological activity of the extract, leading to an early and pronounced cytotoxic response. In summary, the applied biofortification strategy differentiates the cytotoxic properties of the extracts. FeHBED demonstrated the highest efficacy even at low concentrations, whereas FeCl_3_ did not enhance cytotoxicity compared to the non-biofortified extract.

### 2.2. Antioxidant Properties

**Figure 2:**
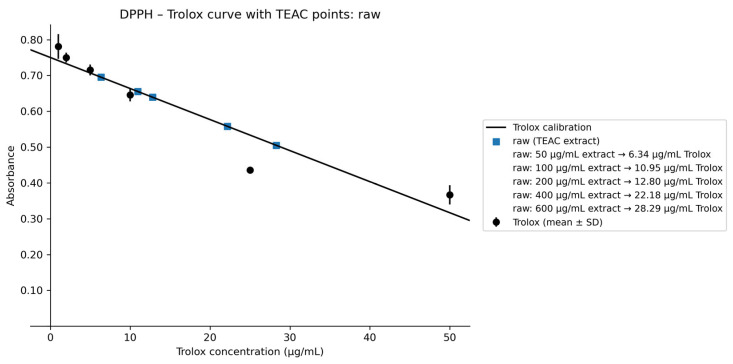
Antioxidant activity of the extract from *Hericium erinaceus* not treated with any form of iron, determined by the DPPH radical scavenging assay and expressed as Trolox equivalents (μg/mL).

The antioxidant properties of *H. erinaceus* extracts were evaluated using the DPPH radical scavenging assay, and the results were expressed as Trolox Equivalent Antioxidant Capacity (TEAC). Both the non-biofortified (raw) extract and the biofortified variants exhibited concentration-dependent antioxidant activity within the range of 50–600 µg/mL, as evidenced by the increase in TEAC values with increasing extract concentration. The non-biofortified extract demonstrated the highest antioxidant activity among the analyzed samples, reaching 28.29 µg/mL Trolox equivalents at a concentration of 600 µg/mL extract (Figure 2).

**Figure 3:**
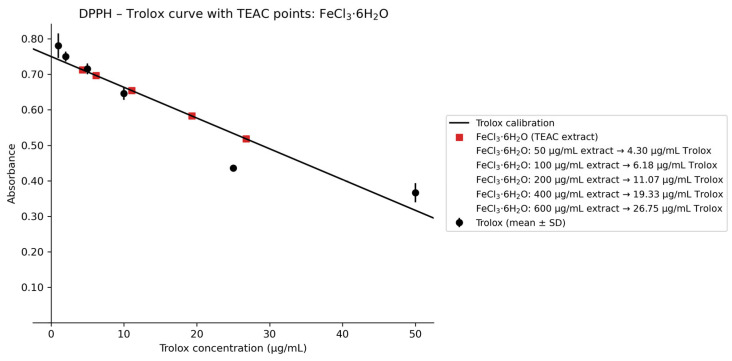
Antioxidant activity of the extract from *Hericium erinaceus* biofortified with FeCl_3_·6H_2_O, determined by the DPPH radical scavenging assay and expressed as Trolox equivalents (μg/mL).

Biofortification with FeCl_3_·6H_2_O resulted in slightly lower TEAC values compared to the non-biofortified extract. Antioxidant activity increased from 4.30 µg/mL Trolox (at 50 µg/mL extract) to 26.75 µg/mL Trolox (at 600 µg/mL extract). Figure 3 shows the results of the experiment.

**Figure 4:**
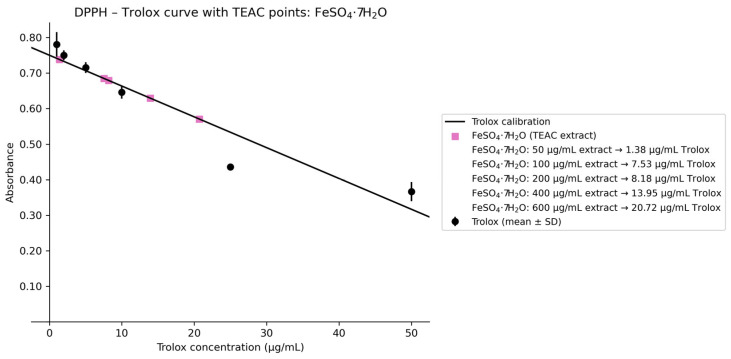
Antioxidant activity of the extract from *Hericium erinaceus* biofortified with FeSO_4_·7H_2_O, determined by the DPPH radical scavenging assay and expressed as Trolox equivalents (μg/mL).

In contrast, the FeSO_4_·7H_2_O-biofortified extract exhibited the lowest radical scavenging capacity among the tested variants, reaching a maximum value of 20.72 µg/mL Trolox at 600 µg/mL extract (Figure 4).

**Figure 5:**
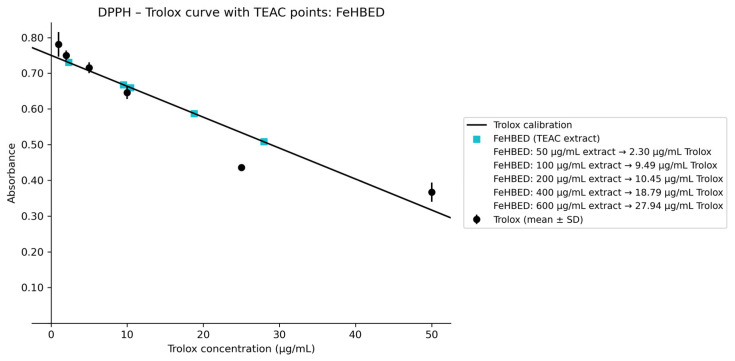
Antioxidant activity of the extract from *Hericium erinaceus* biofortified with FeHBED, determined by the DPPH radical scavenging assay and expressed as Trolox equivalents (μg/mL).

A comparable level of activity to that of the non-biofortified extract was observed for the FeHBED-biofortified extract, which achieved 27.94 µg/mL Trolox equivalents at the highest tested concentration, performing nearly identically to the raw extract (Figure 5).

Overall, all extracts demonstrated clear dose-dependent antioxidant activity and a similar response pattern across the tested concentration range. The highest activity was observed for the non-biofortified and FeHBED-biofortified extracts, whereas biofortification with FeSO_4_·7H_2_O was associated with relatively lower DPPH radical scavenging efficiency.

### 2.3. Cellular Morphology Analysis

**Figure 6:**
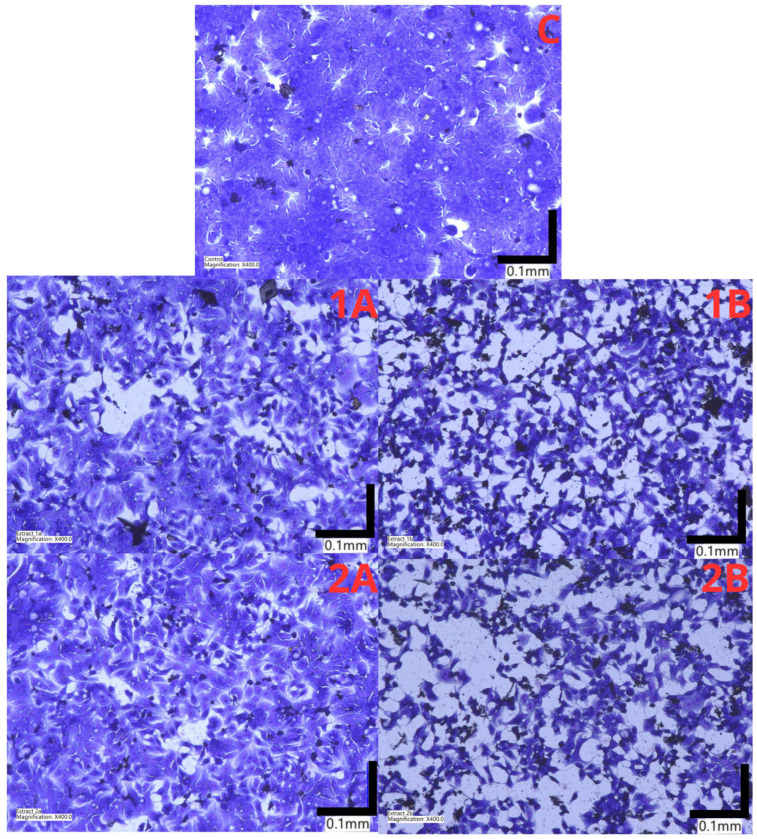
Changes in the morphology and number of HT-29 cells after treatment with *Hericium erinaceus* extracts. Images were acquired using a KEYENCE VHX-X1 microscope at 400× magnification. (**C**) control; (**1A**) non-biofortified extract, 400 µg/mL; (**1B**) non-biofortified extract, 600 µg/mL; (**2A**) *H. erinaceus* biofortified with FeCl_3_·6H_2_O, 400 µg/mL; (**2B**) *H. erinaceus* biofortified with FeCl_3_·6H_2_O, 600 µg/mL.

Microscopic evaluation of HT-29 cells stained with May–Grünwald–Giemsa revealed pronounced, concentration-dependent morphological alterations following treatment with *H. erinaceus* extracts. Control cells (K) displayed typical epithelial morphology characterized by polygonal shape, intact monolayer organization, high confluency, and strong cell–cell adhesion. Treatment with the non-biofortified extract at 400 µg/mL induced moderate morphological changes, including slight reduction in cell density and early signs of cytoplasmic shrinkage. At 600 µg/mL, more evident alterations were observed, such as decreased confluency, partial loss of adherence, and the presence of cells with condensed cytoplasm. Cells treated with extracts from fruiting bodies biofortified with FeCl_3_·6H_2_O exhibited moderate cytotoxic features, particularly at 600 µg/mL, including reduced cell number, increased intercellular spaces, and morphological changes suggestive of early apoptotic-like alterations (Figure 6).

**Figure 7:**
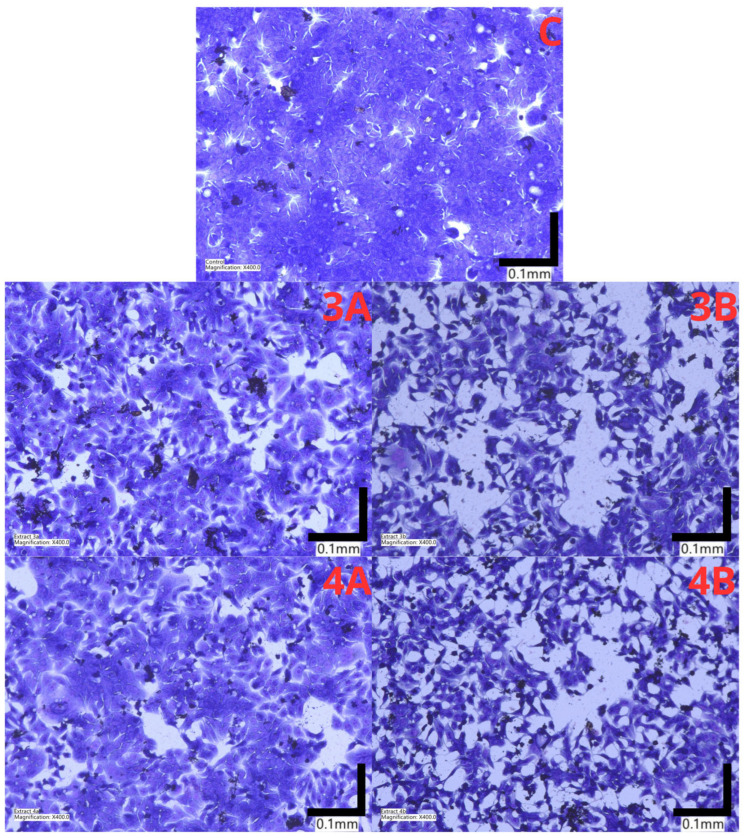
Changes in the morphology and number of HT-29 cells after treatment with *Hericium erinaceus* extracts. Images were acquired using a KEYENCE VHX-X1 microscope at 400× magnification. (**C**) control; (**3A**) *H. erinaceus* biofortified with FeSO_4_·7H_2_O, 400 µg/mL; (**3B**) *H. erinaceus* biofortified with FeSO_4_·7H_2_O, 600 µg/mL; (**4A**) *H. erinaceus* biofortified with FeHBED, 400 µg/mL; (**4B**) *H. erinaceus* biofortified with FeHBED, 600 µg/mL.

More pronounced cytotoxic effects were observed after treatment with extracts biofortified with FeSO_4_·7H_2_O and FeHBED. At 400 µg/mL, a marked reduction in cell density and disruption of the monolayer structure were evident. At 600 µg/mL, cells displayed characteristic apoptotic-like morphology, including cell shrinkage, chromatin condensation, loss of adhesion, and fragmentation of the cell monolayer, indicating enhanced antiproliferative and pro-apoptotic activity (Figure 7).

Overall, May–Grünwald–Giemsa staining confirmed that iron biofortification—particularly with FeSO_4_·7H_2_O and FeHBED—intensified the cytotoxic and morphological effects of *H. erinaceus* extracts on HT-29 cells compared to the non-biofortified and FeCl_3_-treated variants. In addition to qualitative observations, quantitative image analysis revealed a significant, concentration-dependent reduction in cell density. For the FeHBED-biofortified extract at 600 µg/mL, the number of adherent cells decreased by 71% compared to the control, while the percentage of cells displaying clear apoptotic-like features reached 46%. These quantitative data provide statistical support for the superior antiproliferative effect of the chelated iron variant observed in the morphological and cytotoxicity assays.

### 2.4. Expression of Genes Associated with Cancer of the Large Intestine

**Figure 8:**
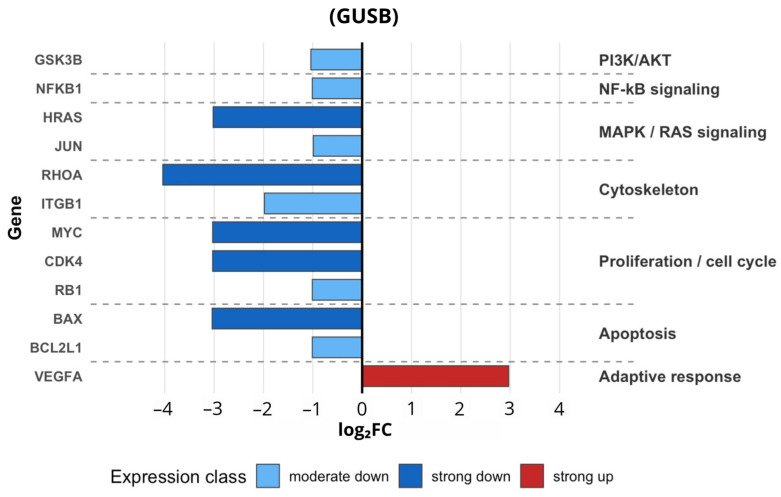
Relative gene expression (log_2_FC) in cells treated with *Hericium erinaceus* extract biofortified with FeHBED (600 µg/mL), determined by RT-qPCR and normalized to the reference gene GUSB. Color coding reflects the magnitude of regulation according to predefined log_2_FC thresholds. From the original panel of 92 genes, 12 were selected for further analysis based on predefined quality and biological relevance criteria. Genes that did not show detectable expression, as well as those with cycle threshold (Ct) values above 37 at any stage of the experiment, were excluded to ensure data reliability. Among the remaining candidates, genes exhibiting the most significant changes in expression relative to the control group were prioritized. As a result, the final set of 12 genes represents those with the most robust and biologically meaningful differences in expression.

The FeHBED-biofortified extract at a concentration of 600 µg/mL was selected for high-throughput molecular profiling because it elicited the maximum biological response in both cytotoxicity and morphological assays. As the highest tested dose, it provided the most robust experimental model to identify the specific signaling pathways and gene regulatory networks responsible for the potent anticancer effects observed in HT-29 cell. Based on the analyses of cytotoxicity, cell viability, morphology, and antioxidant capacity, the extract of *H. erinaceus* biofortified with chelated iron (FeHBED) at a concentration of 600 µg/mL was selected for further investigation of molecular mechanisms in human cancer cells. This variant exhibited the highest anticancer potential among all tested samples. The FeHBED-biofortified extract demonstrated the strongest cytotoxic effect compared to the non-fortified extract and samples treated with other iron forms. At the same time, its antioxidant capacity remained comparable to that of the crude extract. Morphological analysis revealed that, at a concentration of 600 µg/mL, treated HT-29 cells displayed characteristic features including cell shrinkage, chromatin condensation, loss of adhesion, and fragmentation of the cellular monolayer. These observations indicate pronounced antiproliferative activity. Staining with the May–Grünwald–Giemsa method confirmed that biofortification with FeHBED enhanced both cytotoxic and morphological effects of *H. erinaceus* extracts on HT-29 cells compared to non-biofortified variants and those treated with inorganic iron. Gene expression analysis performed using RT-qPCR demonstrated that treatment with the FeHBED-biofortified *H. erinaceus* extract (600 µg/mL) resulted in significant alterations in the expression levels of selected regulatory genes (Figure 8).

Expression levels were presented as log_2_ fold change (log_2_FC) relative to control cells after normalization to the reference gene GUSB. A total of 92 mRNAs associated with human cancer-related molecular pathways were analyzed in both control and treated samples. The selection of specific genes for detailed discussion was based on a combination of statistical magnitude—prioritizing those with a log_2_FC ≥ 0.5—and their established functional roles in the biological processes observed in this study, such as proliferation, programmed cell death, and cellular motility. Among three candidate endogenous control genes (GAPDH, HPRT1, and GUSB), GUSB was selected as the most suitable reference gene due to its lowest variability across experimental conditions, as indicated by minimal standard deviations of Ct values. The strongest upregulation was observed for VEGFA (log_2_FC = 2.97), indicating a substantial increase in its transcription following treatment. Moderate downregulation (log_2_FC between −0.5 and −1.5) was observed for JUN, RB1, NFKB1, BCL2L1, and GSK3B. A stronger decrease in mRNA levels (log_2_FC between −1.5 and −2.99) was noted for ITGB1 (log_2_FC = −1.98). The most pronounced downregulation (log_2_FC < −3) was identified for HRAS, MYC, CDK4, BAX, and RHOA, indicating strong inhibition of their expression.

Overall, the obtained results suggest that the FeHBED-biofortified *H. erinaceus* extract induces a coordinated cellular response involving inhibition of proliferation and cell cycle progression, suppression of pro-survival and inflammatory signaling pathways, and reduction in cellular migratory potential. Simultaneously, activation of stress-related signaling pathways was observed. This integrated molecular response highlights the potential anticancer properties of the tested extract and supports its relevance as a promising candidate for further functional studies.

## 3. Discussion

The above study provides evidence that iron biofortification significantly affects the anticancer activity of *H. erinaceus* extracts against colorectal cancer cells. This was demonstrated by studies of cytotoxicity, antioxidant capacity, morphology, and expression of genes associated with cancer of the large intestine. The results showed clear differences depending on the chemical form of iron. The FeHBED-biofortified extract exhibited the strongest cytotoxic activity. This is the first study comparing different forms of iron used for the biofortification of *H. erinaceus* in the context of colorectal cancer research, highlighting the key role of iron speciation in modulating biological activity. It should be noted that these extracts were additionally subjected to in vitro digestion to better reflect their action in the human body. These results are consistent with previous reports indicating that *H. erinaceus* exhibits anticancer properties, primarily through mechanisms related to oxidative stress and apoptosis [23].

The cytotoxicity of the tested extracts was concentration-dependent, which is consistent with the literature describing the dose-dependent antiproliferative effects of bioactive compounds of fungal origin [24]. A significant observation, however, is the increased cytotoxicity of the FeHBED-biofortified extract, which caused a significant reduction in cell viability even at low concentrations. This effect can be attributed to the increased bioavailability of iron and its more efficient delivery into the cell, characteristic of chelated forms. Iron chelates maintain iron in a soluble and bioavailable form, facilitating its cellular uptake and increasing the pool of intracellular labile iron, which plays a key role in the generation of oxidative stress [25].

Elevated intracellular iron levels promote the formation of highly reactive hydroxyl radicals, leading to damage to proteins, lipids, and DNA. This mechanism is widely recognized as a key factor in the death of cancer cells induced by extracts enriched with metals and natural compounds [26]. The higher cytotoxicity of FeHBED compared to FeSO_4_ and FeCl_3_ can be explained by its greater stability and ability to effectively deliver active iron, thereby inducing oxidative stress.

While the DPPH results demonstrate a high chemical radical-scavenging potential for the FeHBED-biofortified extract in a cell-free system, these findings should not be directly equated with intracellular antioxidant protection. In the complex environment of a tumor cell, the presence of highly bioavailable iron (particularly in the FeHBED form) can shift the role of these bioactive compounds from antioxidants to pro-oxidants, potentially fueling the Fenton reaction and generating reactive oxygen species. Consequently, the observed antioxidant capacity likely represents the chemical reactivity of the extract rather than a definitive intracellular redox mechanism, and the link between DPPH activity and the observed cytotoxic effect remains suggestive rather than causative [27].

The phenolic compounds present in *H. erinaceus* extracts can reduce Fe^3+^ to Fe^2+^, leading to increased production of reactive oxygen species. The FeHBED-biofortified extract may act as a pro-oxidant system within cells, even though it exhibits high antioxidant activity in vitro [25].

Morphological analysis confirms the involvement of oxidative stress-dependent mechanisms of cell death. Cell shrinkage, chromatin condensation, and loss of adhesion are characteristic of apoptosis. Studies show that *H. erinaceus* induces apoptosis via ROS-dependent mitochondrial pathways [24].

The dependence of cytotoxicity on the form of iron also suggests a possible role for iron-dependent cell death mechanisms, such as ferroptosis. Iron chelation may increase the susceptibility of cancer cells to iron-dependent oxidative death pathways. Increased availability of active iron, particularly in the case of FeHBED, may promote lipid peroxidation and activation of ferroptotic pathways, as confirmed by studies showing that iron supplementation enhances ferroptosis in cancer cells [28]. These observations, combined with the strong dependence of the cytotoxic effect on iron speciation, suggest a potential role for iron-dependent cell death mechanisms, such as ferroptosis. However, as specific markers such as GPX4 levels or lipid peroxidation were not evaluated in this study, the involvement of ferroptosis remains a hypothesis that requires further experimental validation. At this stage, our data primarily confirm an apoptotic-like mechanism accompanied by the selective modulation of oncogenic gene expression [29].

The effects of the FeCl_3_-biofortified extract further underscore the importance of using the appropriate form of iron. FeCl_3_ provides Fe^3+^, which is less bioavailable due to rapid hydrolysis and precipitation under physiological conditions, resulting in limited cellular uptake and poor biological activity. These results underscore that not only the presence of iron but also its chemical form critically determines biological effects [30].

It is important to consider that the enhanced anticancer activity of the FeHBED-biofortified extract may not be solely due to iron speciation itself, but rather to a synergy between the bioavailable iron and changes in the composition of bioactive mushroom metabolites. Our previous study demonstrated that FeHBED significantly alters the metabolic profile of *H. erinaceus* during growth. Therefore, the in vitro digestion process likely releases a unique profile of bioaccessible compounds that, in combination with stable chelated iron, target oncogenic pathways more effectively than inorganic iron forms. While the current study focuses on the functional cellular response to these digested extracts, the observed effects likely reflect this multifaceted interaction between iron speciation and fungal secondary metabolism.

The biological effects of biofortified *H. erinaceus* extracts observed in the present study seem to involve multiple interconnected cellular mechanisms affecting proliferation, survival signaling, and stress responses in HT-29 colorectal cancer cells.

These findings are consistent with the gene expression data obtained by RT-qPCR, which revealed the downregulation of several key regulators of cell cycle progression and oncogenic signaling, including HRAS, MYC, and CDK4. HRAS is a proto-oncogene belonging to the RAS family that activates major proliferative pathways such as MAPK/ERK and PI3K/AKT, which are essential for tumor cell growth and survival. Therefore, reduced *HRAS* expression may indicate suppression of proliferative signaling cascades [31]. Similarly, the transcription factor MYC, one of the most important drivers of tumor cell proliferation and metabolic reprogramming, was also downregulated, which is commonly associated with the antiproliferative activity of anticancer compounds [32]. In addition, decreased expression of *CDK4*, encoding a key cyclin dependent kinase controlling the G1–S cell cycle transition, suggests that the extract may induce cell cycle arrest and exert cytostatic effects in HT-29 cells [33]. The inhibitory effect on tumor cell proliferation was accompanied by changes in genes associated with cell migration and invasive potential. Notably, significant downregulation of *RHOA* and *ITGB1* was observed. RHOA plays a critical role in cytoskeleton organization and regulation of cell motility, whereas integrin β1 (ITGB1) mediates adhesion to the extracellular matrix and contributes to tumor cell invasion and metastasis [34]. The suppression of these genes may therefore indicate that the biofortified mushroom extract not only inhibits cell proliferation but may also reduce the migratory and invasive capacity of colorectal cancer cells.

The pronounced activity of the FeHBED-biofortified extract compared to other forms can be attributed to the high stability and water solubility of the HBED chelate. While inorganic iron forms, such as FeCl_3_, are prone to rapid hydrolysis and precipitation in the gastrointestinal environment, the FeHBED complex ensures superior bioavailability and more efficient cellular uptake of iron. This leads to a significant expansion of the intracellular labile iron pool, which is essential for fueling pro-oxidant reactions and subsequent molecular changes. Beyond simple iron delivery, our results suggest an alternative mechanism involving a speciation-dependent synergy: the FeHBED form likely interacts with *H. erinaceus* metabolites (e.g., erinacines and phenols) to create a more potent biological system that selectively downregulates key oncogenes like HRAS and MYC, a response not observed with less bioavailable iron forms.

Interestingly, despite the overall antiproliferative effect, the expression of *VEGFA* was upregulated in treated cells. In colorectal cancer cells, activation of the HIF-1α–VEGFA signaling axis is a well-known response to metabolic stress [35]. HT-29 cells are particularly sensitive to metabolic disturbances, and increased *VEGFA* expression has frequently been reported following exposure to plant- or fungus-derived bioactive compounds that induce oxidative stress or mitochondrial dysfunction [36]. Therefore, the observed increase in *VEGFA* expression may represent a stress-induced adaptive response rather than a direct pro-tumorigenic effect, reflecting the activation of survival mechanisms in response to unfavorable intracellular conditions.

This explanation is supported by the simultaneous modulation of genes involved in pro-survival signaling. Moderate downregulation of *NFKB1* (nuclear factor kappa B subunit 1), encoding a central transcription factor regulating inflammatory signaling and cell survival, may increase the susceptibility of cancer cells to apoptosis [37]. In addition, decreased expression of the anti-apoptotic gene *BCL2L1* (Bcl-xL) suggests reduced anti-apoptotic protection of the cells. Although a decrease in *BAX* expression was also observed, this does not necessarily exclude apoptosis induction, as the activity of BAX is largely regulated at the post-translational level and depends on mitochondrial translocation and interactions with other BCL-2 family proteins [38]. The observed downregulation of BAX mRNA, while seemingly inconsistent with typical apoptosis induction, highlights the complexity of the cellular response to FeHBED-biofortified extracts. It is well established that BAX function is critically controlled by post-translational modifications and mitochondrial translocation, rather than by transcript levels alone. Furthermore, the simultaneous and significant suppression of anti-apoptotic BCL2L1 and pro-survival NFKB1 suggests a shift in the intracellular balance toward programmed cell death. Therefore, the morphological hallmarks observed such as chromatin condensation and cellular fragmentation likely reflect an apoptotic-like pathway triggered by the loss of survival signaling, even in the absence of BAX transcriptional upregulation. The selective modulation of gene expression was further supported by the lack of significant changes in several regulatory genes, including *PTEN*, *MAPK1*, *RAC1*, *CDKN1B*, *CDKN2A*, and *NFKBIA*, suggesting that the extracts did not induce global transcriptional suppression but rather targeted specific signaling pathways associated with tumor cell proliferation, survival, and migration. Furthermore, the lack of significant changes in the expression of several key regulatory genes, such as PTEN and MAPK1, underscores the selective nature of the FeHBED-biofortified extract’s action, indicating that the observed molecular alterations are not a result of non-specific global transcriptional suppression induced by high cytotoxicity.

The biological activity observed in this study may be attributed to the diverse bioactive compounds present in *H. erinaceus*. This medicinal mushroom contains numerous secondary metabolites, polysaccharides, phenolic compounds, and sterols, many of which have been reported to exert antioxidant and anticancer activities. Previous studies have shown that metabolites derived from *H. erinaceus* can induce oxidative stress, apoptosis, and cell cycle arrest in cancer cells [9]. Iron is an essential cofactor in numerous redox reactions and may promote the generation of reactive oxygen species through Fenton-type reactions, which catalyze the conversion of hydrogen peroxide into highly reactive hydroxyl radicals [39]. The resulting increase in ROS levels may contribute to cellular stress and stabilization of HIF-1α, which in turn stimulates *VEGFA* transcription. This mechanism could explain the simultaneous observation of *VEGFA* upregulation and reduced proliferation of HT-29 cells [40].

It should be acknowledged that the present study was conducted using a single colorectal cancer model (HT-29), which may limit the generalizability of these findings owing to the unique genetic and metabolic characteristics of this cell line. Although the selective modulation of gene expression observed via RT-qPCR suggests a targeted effect rather than general toxicity, further studies involving additional cancer models (such as HCT116 or SW480) and normal colon epithelial cells are necessary. Such research will be crucial to definitively confirm the selectivity of biofortified *H. erinaceus* extracts and to further establish their translational potential as functional food components.

Taken together, the results suggest that biofortified *H. erinaceus* extracts exert a multifaceted biological effect on colorectal cancer cells, involving inhibition of proliferative signaling pathways, reduction in migration-related gene expression, and activation of stress-related adaptive responses. The combined action of mushroom-derived bioactive metabolites and iron-mediated redox modulation may therefore contribute to the observed cytostatic and potentially anti-metastatic effects in HT-29 cells.

## 4. Materials and Methods

### 4.1. Biofortification

The initial characteristics of the biofortified fruiting bodies, including iron accumulation levels and metabolic profiles, were established in our previous study (https://doi.org/10.1016/j.foodchem.2025.147238), ensuring the reproducibility and standardization of the material used for the current extraction and digestion processes. The aforementioned study detailed each stage of the process, including the preparation of microorganisms and spawn, substrate preparation, cultivation conditions and the preparation of carpophores [15]. For the biofortification process, three iron compounds were used as enrichment agents: iron(III) chloride hexahydrate (FeCl_3_·6H_2_O), iron(II) sulfate heptahydrate (FeSO_4_·7H_2_O), and bis(2-hydroxybenzyl)ethylenediamine diacetic acid ferric potassium complex (FeHBED) (Merck KGaA, Darmstadt, Germany). Each compound was introduced at a concentration corresponding to 10 mM Fe. A non-supplemented system without the addition of iron, served as the control.

### 4.2. Simulated In Vitro Gastrointestinal Digestion of Hericium erinaceus Extract

In vitro digestion was performed as described in our previously work [41]. The digested samples were centrifuged (10,700× *g* for 30 min at 4 °C) and frozen at 80 °C for 24 h. Next, lyophilization was carried out in a Labconco freeze dryer (Labconco, Kansas City, MO, USA).

### 4.3. Cell Cultures

Experiments were performed using the human colorectal adenocarcinoma cell line HT-29 (ATCC^®^ HTB-38™). The cells were maintained in RPMI 1640 medium supplemented with either 10% or 2% fetal bovine serum (FBS). The culture medium was enriched with antibiotics, specifically penicillin (100 U/mL) and streptomycin (100 μg/mL). Cells were incubated under standard culture conditions at 37 °C in a humidified atmosphere containing 5% CO_2_.

### 4.4. Samples Preparation

The lyophilized samples were accurately weighed and subsequently dissolved in a mixture of RPMI 1640 culture medium and DMSO at a 1:1 ratio. Stock solutions were prepared at a concentration of 100 mg/mL. At the highest tested sample concentration, the DMSO content did not exceed 0.25%. According to our earlier findings, this level of DMSO does not exert cytotoxic effects within the experimental time frame applied in this study.

### 4.5. Cytotoxicity

#### MTT Assay

The MTT assay was used to evaluate cell viability and proliferative capacity. This method is based on the assessment of mitochondrial oxidoreductase activity. In metabolically active cells, the yellow tetrazolium salt (MTT) is reduced by mitochondrial succinate dehydrogenase to insoluble purple formazan crystals, resulting in a visible color shift from yellow to purple. Cells were seeded into 96-well plates at a density of 1 × 10^5^ cells/mL. After 24 h, the culture medium was aspirated and replaced with fresh medium containing appropriate concentrations of the in vitro digested *H. erinaceous* extract. All biofortification variants were evaluated at 50, 100, 200, 400, and 600 µg/mL. Following a 24 h incubation period, either in the presence or absence of the tested extract, MTT solution (5 mg/mL in PBS) was added, and the plates were incubated for an additional 3 h to allow for formazan formation. The reaction was terminated by adding 10% SDS prepared in 0.01 N HCl to solubilize the crystals. After overnight incubation (24 h), absorbance was measured at 570 nm using a Varioskan™ LUX multimode microplate reader (Thermo Fisher Scientific, Waltham, MA, USA). The measured absorbance values, corresponding to the intensity of the purple coloration, were directly proportional to the number of viable cells [42]. The assay was conducted in three independent experiments, with six technical replicates per concentration in each run.

### 4.6. Antioxidant Properties

#### DPPH Method

The antioxidant activity of the tested compounds and preparations was evaluated using the DPPH radical scavenging assay. This method measures the ability of antioxidants to neutralize the stable DPPH radicals. The reaction involves the donation of a hydrogen atom to the nitrogen-centered free radical of DPPH, leading to the formation of the corresponding hydrazine. This process results in a decrease in absorbance and a visible color change from purple to yellow, associated with the reduction in DPPH [43].

The analysis was performed in 96-well microplates. Methanolic solutions of the tested samples and Trolox were prepared at appropriate dilutions. All biofortification variants were evaluated at concentrations of 50, 100, 200, 400, and 600 µg/mL. Trolox was used as the reference antioxidant (positive control), and methanol was used as the negative control. Subsequently, 100 µL of each dilution (samples, Trolox, or methanol) was added to the wells, followed by 100 µL of DPPH solution prepared in methanol at a concentration of 0.2 mg/mL. After incubation for 10 min at room temperature, absorbance was measured at 515 nm using a Varioskan™ LUX multimode microplate reader (Thermo Fisher Scientific, Waltham, MA, USA). The antioxidant capacity measurements were performed in three independent experimental series.

### 4.7. Cellular Morphology Analysis

#### May–Grünwald–Giemsa Staining

This procedure was employed to evaluate morphological alterations in cells following exposure to the tested substances. Cells were seeded into 24-well plates at a density of 5 × 10^5^ cells/mL (1 mL per well). After 24 h of incubation in the presence or absence of the examined extracts, the culture medium was carefully aspirated, and the cells were subjected to May–Grünwald staining. All biofortification variants were evaluated at concentrations of 50, 100, 200, 400, and 600 µg/mL. Specifically, 1 mL of May–Grünwald solution was added to each well and incubated for 3 min at room temperature. Subsequently, 1 mL of deionized water was added, and after an additional 3 min incubation, the staining solution was removed. The wells were gently rinsed with deionized water and then stained with Giemsa solution diluted 1:20 for 30 min at room temperature. Following staining, the dye was discarded, the wells were washed again with 1 mL of deionized water and the plates were air-dried. Cellular morphology was documented using a KEYENCE VHX-X1 microscope (KEYENCE Corporation, Osaka, Japan).

### 4.8. Expression of Genes Associated with Cancer of the Large Intestine

#### 4.8.1. RNA Isolation and Genomic DNA Removal from RNA Preparations

Total RNA was isolated from HT-29 cells cultured in RPMI medium supplemented with 2% fetal bovine serum (FBS), as well as from cells grown in the same medium supplemented with *H. erinaceus* extract biofortified with FeHBED at a final concentration of 600 µg/mL. Following trypsinization, the cells were washed with phosphate-buffered saline (PBS) and stored at −70 °C until further processing.

RNA was extracted using the GeneJET RNA Purification Kit (Thermo Scientific) according to the manufacturer’s instructions. Briefly, 5 × 10^6^ cells were lysed in 600 µL of lysis buffer supplemented with β-mercaptoethanol. The samples were mixed briefly, and 360 µL of ethanol was added. The lysates were then transferred to RNA purification columns and centrifuged at 12,000× *g*. After sequential washing steps with the provided wash buffers, the RNA was eluted using nuclease-free water.

Residual genomic DNA was removed by DNase I treatment (A&A Biotechnology, Gdynia, Poland), using 1 U of DNase per 1 µg of RNA. The digestion was carried out at 37 °C for 30 min and terminated by adding EDTA to a final concentration of 5 mM. DNase was subsequently inactivated by incubation at 75 °C for 10 min.

RNA concentration and purity were assessed spectrophotometrically using a NanoDrop 2000 instrument (Thermo Fisher Scientific, Wilmington, DE, USA). The purified RNA was subsequently used for complementary DNA (cDNA) synthesis [44].

#### 4.8.2. cDNA Synthesis and Quantitative Real-Time PCR

Complementary DNA (cDNA) was synthesized from 1 µg of total RNA using the RevertAid First Strand cDNA Synthesis Kit (Thermo Scientific) following the manufacturer’s protocol. Reverse transcription was performed at 42 °C for 60 min, and the reaction was terminated by heating the samples at 70 °C for 5 min. The resulting cDNA was used directly as a template for quantitative real-time PCR (qPCR).

Relative mRNA expression analysis was carried out using quantitative real-time PCR on a StepOnePlus™ Real-Time PCR System (Thermo Fisher Scientific). Amplification reactions were performed with TaqMan™ Fast Advanced Master Mix in combination with a predesigned TaqMan™ Array Plate (Human Molecular Mechanisms of Cancer). The array included 92 assays targeting genes involved in molecular mechanisms of cancer, particularly those associated with p53 signaling pathways, as well as four candidate endogenous control genes. The PCR cycling conditions were as follows: initial incubation at 50 °C for 2 min, enzyme activation at 95 °C for 20 s, followed by 40 cycles of denaturation at 95 °C for 1 s and annealing/extension at 60 °C for 20 s. Gene expression was calculated with GUSB used as the reference gene. In the first step, ΔCt values were determined as the difference between the Ct value of the target gene and the Ct value of the reference gene. Subsequently, ΔΔCt values were calculated as the difference between the ΔCt of the experimental sample and the ΔCt of the control sample. Based on these values, fold change in gene expression was determined using the formula 2^−ΔΔCt^. Additionally, log_2_FC values were calculated. Positive log_2_FC values were interpreted as upregulation, negative values as downregulation, and values close to zero as indicating no significant change relative to the control. The experiments were performed in three independent biological replicates (*n* = 3).

### 4.9. Statistical Analysis

All analyses were performed using the Python (version 3.12; Python Software Foundation, Wilmington, DE, USA) programming environment. Data processing and preparation were conducted with the Pandas library. The Trolox standard curve was fitted using linear regression with the ordinary least squares (OLS) method implemented in the statsmodels package. The significance of the calibration model was evaluated based on the coefficient of determination (R^2^), the *p*-value, and the standard error of the slope.

The Trolox Equivalent Antioxidant Capacity (TEAC) for the analyzed samples was calculated by substituting the mean absorbance values into the linear regression equation of the calibration curve. The standard deviation of TEAC was determined using error propagation for a linear function, according to the following formula:

(1) $${\mathrm{SD}}_{\mathrm{TEAC}}\;=\;\frac{{SD}_{abs}}{\left|a\right|}$$

where *a* denotes the slope of the calibration curve.

Differences between groups were assessed using one-way analysis of variance (ANOVA), with a linear model fitted by the OLS method in statsmodels. The significance of the factor effect was tested at the *α* = 0.05. Post hoc comparisons were performed using Tukey’s Honestly Significant Difference (HSD) test to identify pairs of groups that differed significantly from each other. All experiments were performed in at least three independent replicates (*n* = 3), and the results are presented as the mean ± standard deviation (SD) of these independent trials. Data visualizations were generated using Matplotlib (version 3.11.0; Matplotlib Development Team), and statistical plots were prepared using Seaborn (version 0.13.2; Seaborn Development Team). The analysis script was executed from the command line using the Aargparse module.

## Data Availability

The data presented in this study are available on request from the corresponding author.
